# *Marsdeniayarlungzangboensis* (Apocynaceae, Asclepiadoideae), a new species from Xizang, China

**DOI:** 10.3897/phytokeys.130.34152

**Published:** 2019-08-29

**Authors:** Cheng Liu, Ji-Dong Ya, Yun-Hong Tan, Hua-Jie He, Gui-Jun Dong, De-Zhu Li

**Affiliations:** 1 Germplasm Bank of Wild Species, Kunming Institute of Botany, Chinese Academy of Sciences, Kunming, 650201, Yunnan, China Kunming Institute of Botany, Chinese Academy of Sciences Kunming China; 2 Center for Integrative Conservation, Xishuangbanna Tropical Botanical Garden, Chinese Academy of Sciences, Menglun, Mengla, 666303, Yunnan, China Xishuangbanna Tropical Botanical Garden, Chinese Academy of Sciences Yunnan China; 3 South Asia Biodiversity Research Institute, Chinese Academy of Sciences, Yezin, Nay Pyi Taw, 05282, Myanmar South Asia Biodiversity Research Institute, Chinese Academy of Sciences Nay Pyi Taw Myanmar; 4 The Administration of Nature Reserve, Forestry Bureau of Linzhi Prefecture, Linzhi, 860000, Xizang, China Forestry Bureau of Linzhi Prefecture Linzhi China

**Keywords:** *Marsdenia*, *Marsdeniayarlungzangboensis*, new species, China

## Abstract

*Marsdeniayarlungzangboensis* (Apocynaceae, Asclepiadoideae), a new species from Motuo County, southeastern Xizang of China, is described and illustrated. It is morphologically similar to *M.medogensis*, *M.tenii* and *M.yuei*, the major differences between the new species and the morphological relatives are outlined and discussed. A diagnostic key to the new species and its closely related species in China is provided.

## Introduction

*Marsdenia* R. Brown (1810: 460) (Apocynaceae, Asclepiadoideae, Marsdenieae) (Endress and Bruyns 2000, Endress et al. 2014) was established in 1810 and was named in honor of the plant collector William Marsden (1754–1836), who was the Secretary of the Admiralty from 1795. The genus comprises around 100 species, mainly from tropical and subtropical regions, particularly in Asia, Africa and the Americas (Tsiang and Li 1977, Li et al. 1995, Stevens and Juárez-Jaimes 1999, Endress and Bruyns 2000, Stevens 2009). In the revision of *Marsdenia* in Asia, Malesia, Australia and Papuasia (Forster 1995a, 1995b), some species of *Dregea* E. Meyer, *Gymnema* R. Brown, *Dischidanthus* Tsiang and *Jasminanthes*Blume are subsumed into *Marsdenia*. However, these taxonomic treatments are not fully supported by the current molecular phylogenies (Endress et al. 2014). Some new taxa of this genus have been discovered and described in recent years (Stevens and Juárez-Jaimes 1999, Juárez-Jaimes and Alvarado-Cárdenas 2010, Rapini and Pereira 2011, Fernando and Rodda 2013, Jaimes 2015, Jaimes and Pérez 2015, Jaimes and Saynes 2015, Carnevali et al. 2016, Stevens et al. 2016, Santo et al. 2018a, 2018b). According to the treatment of *Marsdenia* in Flora of China, there are 25 species recognized in China and mainly distributed in the east, south and southwest provinces (Tsiang and Li 1977, Li et al. 1995).

In 2016, we collected an unknown species of Apocynaceae during fieldwork in Motuo County, southeastern Xizang, China. This species was identified as a member of *Marsdenia* by characterizing woody vines with umbelliform inflorescences, campanulate corollas with fleshy corona attached to gynostegium and the erect pollinia attached to the caudicles at the base (Tsiang and Li 1977, Li et al. 1995). After careful comparisons of diagnostic morphological and anatomical features of the closely related species from China and adjacent regions (Hooker 1885, Yoganarasimhan and Subramanyam 1976, Tsiang and Li 1977, Li et al. 1995, Forster 1995a, 1995b, Fernando and Rodda 2013), we concluded that the species is new to science and thus describe and illustrate it hereby. Its morphological characters are compared with the closely related species including *M.medogensis* P. T. Li (1985), *M.tenii* M. G. Gilbert & P. T. Li (Gilbert et al. 1995), *M.yuei* M. G. Gilbert & P. T. Li (Gilbert et al. 1995).

## Materials and methods

Vouchers of *Marsdeniayarlungzangboensis* were collected from Motuo County, Xizang of China. The photographs and phenology data were obtained during the field expeditions.

Morphological observations and measurements of the new species were carried out based on living plants and dry specimens. The morphology of opened corolla, opened calyx, gynostegium and staminal corona, pistil, pollinarium were observed by using a Keyence VHX-700F Digital Microscope (Keyence, Osaka, Japan) and based on dry specimens. All morphological characters are described according to the terminology presented by Li et al. (1995).

## Taxonomic treatment

### Marsdenia yarlungzangboensis

Taxon classificationPlantaeGentianalesApocynaceae

C.Liu, J.D.Ya & Y.H.Tan sp. nov.

B9EEF9BCBD18560BAAE5766F57295AF9

urn:lsid:ipni.org:names:77201389-1

Figs 1 , 2

#### Diagnosis.

*Marsdeniayarlungzangboensis* is morphologically similar to *M.medogensis*, *M.tenii* and *M.yuei*, but differs from *M.medogensis* in inflorescences pubescent; sepals suborbicular; corolla yellow, throat red and densely pilose, shorter lobes and reddish outside, apex emarginate, margin reddish and ciliate; corona lobes triangular, to base of anthers; stigma head hemispherical; and differs from *M.tenii* in leaf blades elliptic; sepals suborbicular; corolla yellow, throat red, lobes reddish outside, margin reddish; stigma head hemispherical, conspicuously exserted from anther appendages and corolla tube; and also differs from *M.yuei* in leaf blades elliptic; inflorescences unbranched and pubescent; sepals suborbicular; corolla yellow, throat red and densely pilose; lobes ovate, apex emarginate; corona lobes to base of anthers.

#### Type.

CHINA. Xizang: Motuo County, Renqinbeng, on margins of the subtropical evergreen broad-leaved forest, 29°20'08.59"N, 95°21'38.42"E, 1848 m a.s.l., 15 Nov 2016, in flowering, *C. Liu, J.D. Ya, H.J. He & C.H. Li 16CS11914* (holotype: KUN!, isotype: KUN!)

#### Description.

Lianas woody, up to 10 m. Stems pale gray, sap white. Branchlets glabrous or distal parts minutely puberulent. ***Leaves*** opposite, petiole 1.5–2.5 cm, puberulent; blades elliptic, 7–12 × 3.5–6 cm, papery, glabrescent or sparsely hairy and denser along veins adaxially, base rounded or shallowly cordate, apex acuminate, margin entire, revolute, abaxially pale; lateral veins 4 or 5 pairs. ***Inflorescences*** umbel-like or with several umbel-like cymules along unbranched rachis, 3–7 cm, rachis at least 1.5 cm; peduncle 1.5–6 cm, pubescent; pedicel 5–7 mm, pubescent. ***Sepals*** suborbicular, pubescent outside, ca. 4 × 3–4 mm, ciliate, basal glands 5. ***Corolla*** yellow, campanulate, 1–1.5 cm in diam., glabrous outside, pubescent inside; tube ca. 5 mm, glabrous outside; throat red and densely pilose; lobes ovate and reddish outside, twisted to the right, 5–6 × 3–4 mm, apex emarginate, margin reddish and ciliate. ***Corona*** lobes triangular, fleshy, to base of anthers, almost flat. Anther appendages oblong, apex membranous; ***Pollinia*** 2 per pollinarium, erect, reniform. Ovary glabrous, ca. 2 mm, 2-carpelled, free. Stigma head hemispherical, conspicuously exserted from anther appendages and corolla tube. Follicles and seeds not seen.

**Figure 1.**
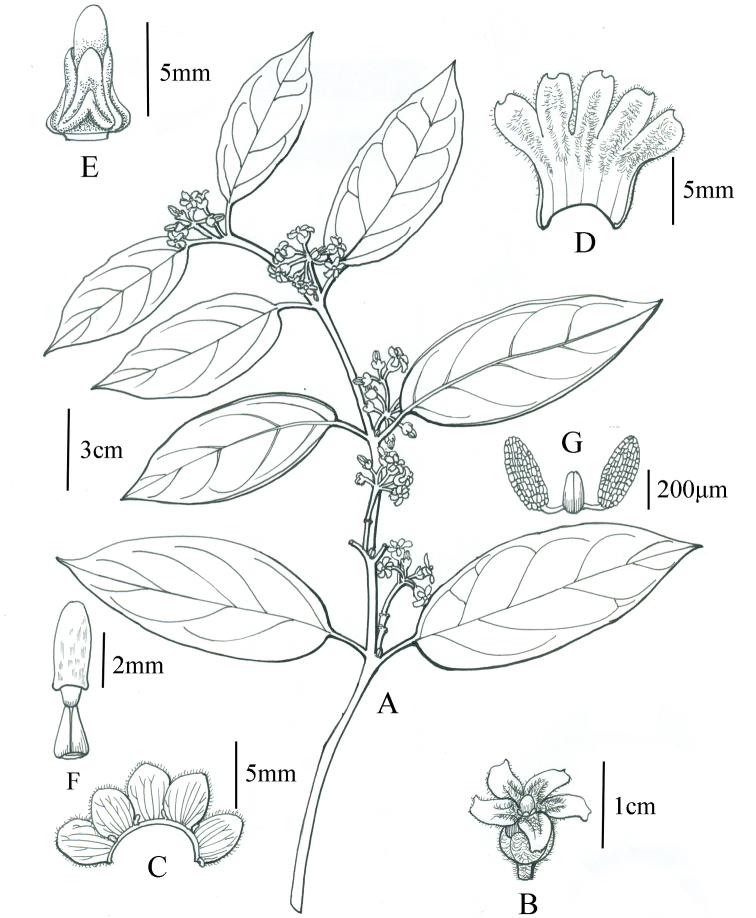
*Marsdeniayarlungzangboensis* C.Liu, J.D.Ya & Y.H.Tan **A** habit **B** flower (lateral view) **C** opened calyx **D** opened corolla **E** gynostegium and staminal corona **F** pistil **G** pollinarium.

**Figure 2.**
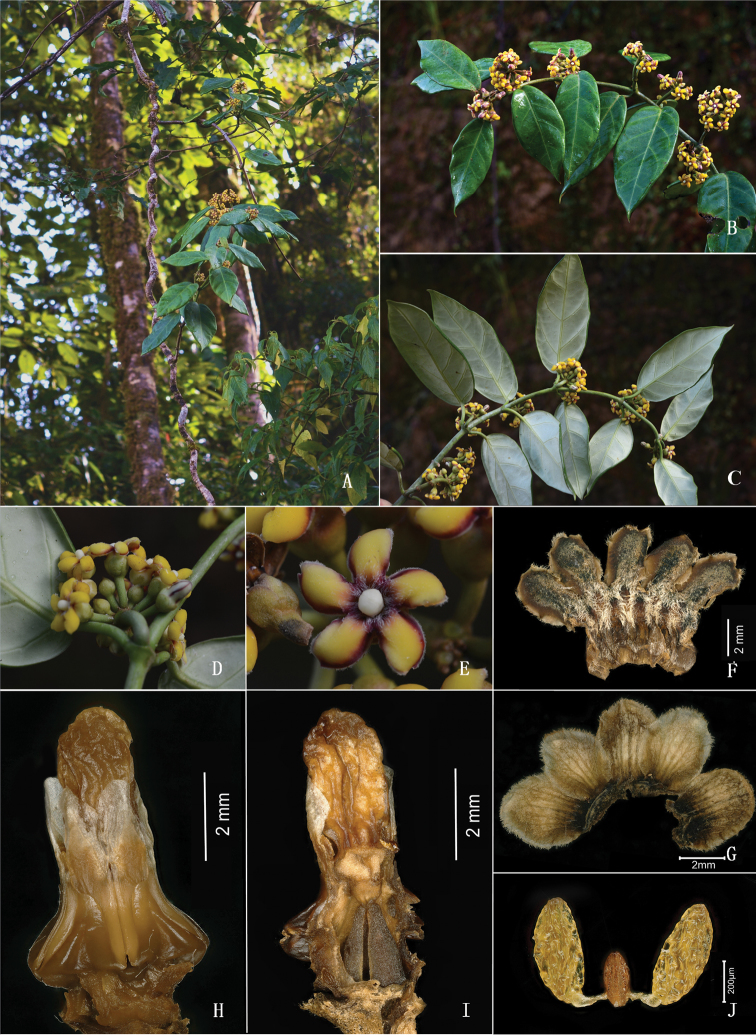
*Marsdeniayarlungzangboensis* C.Liu, J.D.Ya & Y.H.Tan **A** habit **B** inflorescences and adaxial leaf surface **C** abaxial leaf surface **D** flower (lateral view) **E** flower (front view, showing hairy throat) **F** opened corolla **G** opened calyx **H** gynostegium and staminal corona **I** pistil **J** pollinarium. Photo credit: Cheng Liu (**A–E**) and Lian-Yi Li (**F–J**).

#### Phenology.

Flowering from November to December.

#### Distribution and habitat.

*Marsdeniayarlungzangboensis* grows at the margins of subtropical evergreen broad-leaved forest with main community types of *Castanopsisechinocarpa* J. D. Hooker & Thomson ex Miquel and *Quercusgambleana* A. Camus, Renqinbeng, Motuo County, Xizang, China, at an elevation of 1800–2100 m.

#### Etymology.

The specific epithet ‘yarlungzangboensis’ is derived from the type locality, Yarlung Zangbo Grand Canyon, Motuo County, southeast Xizang, China.

#### Vernacular name.

Yǎ Lǔ Zàng Bù Niú Nǎi Cài (Chinese pronunciation); 雅鲁藏布牛奶菜 (Chinese name).

#### Discussion.

Based on the larger gynandrium, corolla tube almost equal in length to gynostegium and with umbel-like cymules along unbranched rachis, *Marsdeniayarlungzangboensis* belongs to Marsdeniasect.Ruehssia (Karst.) Fourn. (Tsiang and Li 1977). Morphologically, it is similar to *M.medogensis*, *M.tenii* and *M.yuei* in terms of habit and floral morphology, but can be distinguished from *M.medogensis* in inflorescences pubescent (vs. glabrous); sepals suborbicular (vs. ovate); corolla yellow (vs. white), throat red and pilose (vs. glabrous), apex emarginate (vs. rounded), margin reddish and ciliate; corona lobes triangular (vs. oblong), to base of anthers (vs. as long as anther appendages); stigma head hemispherical (vs. discoid, convex). It also differs from *M.tenii* in its stems glabrous or distal parts minutely puberulent (vs. densely yellow-brown tomentose); leaf blades elliptic (vs. oblong-ovate); sepals suborbicular (vs. elliptic); corolla yellow, throat red and pilose, lobes reddish outside (vs. yellowish white, throat pilose with retrorse hairs); stigma head hemispherical (vs. 2-cleft, conical), conspicuously exserted from anther appendages and corolla tube (vs. equalling anther appendages). Beyond that, it can be distinguished from *M.yuei* in leaf blades elliptic (vs. ovate); inflorescences unbranched, pubescent (vs. branched, glabrous); sepals suborbicular (vs. rounded); corolla yellow (vs. white), throat red and densely pilose (vs. glabrous); lobes ovate, apex emarginated (vs. oblong-obovate, apex rounded); corona lobes to base of anthers (vs. as long as anther appendages). The detailed characters amongst the three related species are provided in Table 1.

**Table 1.** Diagnostic character differences amongst *Marsdeniayarlungzangboensis*, *M.medogensis*, *M.tenii* and *M.yuei.*

Character	*M.yarlungzangboensis*	*M.medogensis*	*M.tenii*	*M.yuei*
Stems	stems pale gray, glabrous or distal parts minutely puberulent	stems pale gray, nodes pilose	stems densely yellow-brown tomentose	stems glabrous except for flowers
Leaves	petiole 1.5–2.5 cm; blades elliptic, 7–12 × 3.5–6 cm, base rounded or shallowly cordate, abaxially pale, lateral veins 4 or 5 pairs	petiole 1–1.6 cm; blades oblong, 10–11 × 2–3 cm, base rounded, lateral veins 7 or 8 pairs	petiole to 4 cm; blades oblong-ovate, to 12.5 × 7.5 cm, base cordate, lateral veins ca. 5 pairs;	petiole ca. 4 cm; blades ovate, ca. 9.5 × 5.8 cm, base shallowly cordate, lateral veins 4 or 5 pairs
Inflorescences	with several umbel-like cymules along unbranched rachis, pubescent; rachis to at least 1.5 cm	umbel-like, glabrous 4–8–flowered	with several umbel-like cymules along unbranched rachis; rachis to at least 2 cm	umbel-like, up to 9–flowered
Peduncle and Pedicel	peduncle 1.5–6 cm, pedicel 5–7 mm, pubescent	peduncle 4–4.5 cm, pedicel 2–2.5 cm, glabrous	peduncle to 3 cm, pedicel ca. 5 mm	peduncle ca. 1.5 cm, pedicel to 9 mm
Sepals	suborbicular, pubescent outside, ca. 4 × 3–4 mm, basal glands 5	ovate, ca. 4 × 2 mm	elliptic, ca. 3 × 2 cm	rounded, ca. 3 × 2.5 mm, finely appressed puberulent
Corolla	yellow, ca. 1–1.5 cm in diam., throat red and densely pilose; lobes ovate, ca. 5–6 × 3–4 mm, apex emarginate, margin reddish and ciliate	white, 1.5–2 cm in diam., throat glabrous; lodes broadly ovate, ca. 9 × 9 mm, apex rounded	yellowish white, ca. 6 mm, throat pilose with retrorse hairs, lobes ca. 3.5 × 2.5 mm, lobes densely appressed tomentose in center	white, glabrous except for sparsely ciliate margin, lobes oblong-obovate, ca. 5.5 × 2.5–3.2 mm, apex rounded
Corona lobes	triangular, to base of anthers	oblong, as long as anther appendages	to base of anthers	narrowly triangular, as long as anther appendages
Stigma head	hemispherical, conspicuously exserted from anther appendages and corolla tube	discoid, convex, slightly exserted from anther appendages	2-cleft, conical, equalling anther appendages	hemispherical, conspicuously exserted from anther appendages and corolla tube

According to field surveys, this new species is only found in the type locality Renqinbeng, Motuo County, Xizang. This area is one of China’s biodiversity hotspots and consists of a diverse series of ecosystems from subtropical broad-leaved forests to alpine meadows above the tree line with an altitudinal range of 150–6000 m above sea level. Plant diversity is also poorly studied in this area, and some new taxa have been discovered in recent years. In order to better understand and conserve the biodiversity in this area, more extensive investigations are needed in the future.

##### A diagnostic key to the new species and its closely related species in China

**Table:** 

1a	Corolla tube longer than lobes	***M.sinensis***
1b	Corolla tube shorter than lobes	**2**
2a	Corolla lobes 9–12 mm	**3**
3a	Leaf blade 5.5–10 cm wide; inflorescences 7–15 cm; corolla interior pubescent; sepals ca. 8 × 6 mm	***M.koi***
3b	Leaf blade 2–3 cm wide; inflorescences 4–4.5 cm; corolla glabrous; sepals 3–4 × 2–2.5 mm	***M.medogensis***
2b	Corolla lobes 3–7 mm	**4**
4a	Base of leaf blade rounded to truncate; inflorescences unbranched	**5**
5a	Plants tomentose; corolla white, 6–8 mm, stigma head 2-cleft, conical, equalling anther appendages	***M.tenii***
5b	Plants puberulent; corolla yellow, 1–1.5 cm, stigma head hemispherical, conspicuously exserted from anther appendages and corolla tube	***M.yarlungzangboensis***
4b	Base of leaf blade cordate; inflorescences usually obviously branched	**6**
6a	Peduncle to 16 cm; corolla interior pilose; corona lobes shorter than corolla tube	***M.hainanensis***
6b	Peduncle ca. 1.5 cm; corolla interior glabrous; corona lobes longer than corolla tube	***M.yuei***

## Supplementary Material

XML Treatment for Marsdenia yarlungzangboensis
